# Penile resurfacing using a reverse bilateral anterior scrotal artery flap: A case report of penile skin defects following circumcision

**DOI:** 10.1097/MD.0000000000018106

**Published:** 2019-12-10

**Authors:** Qing-Guo Gao, Wenrui Qu

**Affiliations:** a Department of Hand surgery, the second Hospital of Jilin University, Changchun; b Department of Plastic and Reconstructive Microsurgery, China-Japan Union Hospital of Jilin University, Changchun, Jilin province, China.

**Keywords:** circumcision, penis, reconstruction, scrotal flap

## Abstract

**Rationale:**

Circumcision is one of the most frequently used surgical procedures worldwide. Extensive penile skin defects, which can occur as a rare but severe complication of circumcision, are serious and frustrating problems for patients who experience them. Procedures for correcting these problems can pose a challenge to plastic surgeons in the clinic.

**Patient concerns:**

A 31-year-old man was admitted to our care with an extensive defect of the penile skin caused by a circumcision performed 20 days previously.

**Primary diagnoses:**

Infection, necrosis, and defects of the penile skin.

**Interventions:**

A reverse bilateral anterior scrotal flap was used to correct complete penile skin loss following debridement of the infected and necrotic tissue.

**Outcomes:**

The patient experienced no complications during the 10-year follow-up period. The patient reported normal erectile function and the ability to perform intercourse.

**Lessons:**

The reverse bilateral anterior scrotal artery flap is suitable for repairing skin defects of the penis and allows for satisfactory cosmetic and functional improvement following defects of the penile skin.

## 1 Introduction

Circumcision is one of the most commonly applied surgical procedures.^[1]^ The procedure is simple and generally has few complications when performed by an experienced surgeon. When complications do occur, the majority of them are minor and can be corrected easily.^[2]^ However, on rare occasions, more serious complications can arise, including extensive defects of the penile skin. These defects often require surgical correction, resulting in a significant financial burden on the health care system and risk to the patient. Due to the complexity of the penis, repair and reconstruction present a formidable challenge anatomically, functionally and cosmetically.^[3]^ The aim of penile reconstructive surgery is to improve the cosmetic appearance of the penis to a point that is acceptable for the patient and to restore functionality of the penis, allowing the patient to recover normal erectile function and the ability to perform intercourse.^[4]^ The full-thickness skin graft (FTSG) is a commonly used and effective tool in the management of severe penile skin defects; however, the use of FTSG can lead to contracture of the penile skin and extensive scarring.^[5,6]^ Few studies in the available literature have reported on the durability and viability of the local flaps used to treat defects of the penile skin. The key to reconstruction is to choose tissue for a flap that has properties similar to the tissue of the repair site to achieve acceptable cosmetic and functional results. A scrotal skin flap, with sufficient blood supply and plenty of nerve endings, could, therefore, provide excellent therapeutic effects when used in cases of penile skin defects. Here, we demonstrate the use of a reliable and effective reconstruction method using a reverse bilateral scrotal flap to repair extensive defects of the penile skin through a one-stage operation.

## 2 Case report

**Figure 1:**
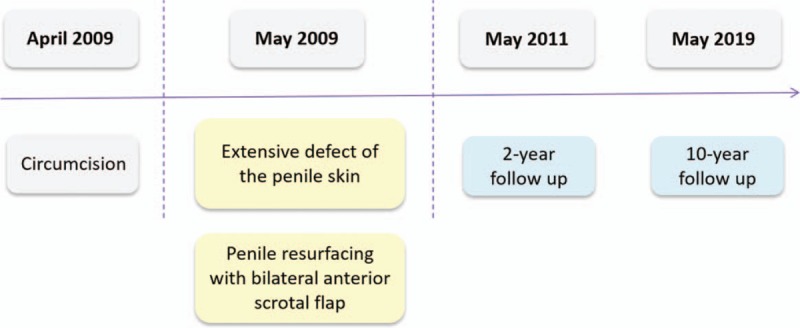
Patient timeline.

**Figure 2:**
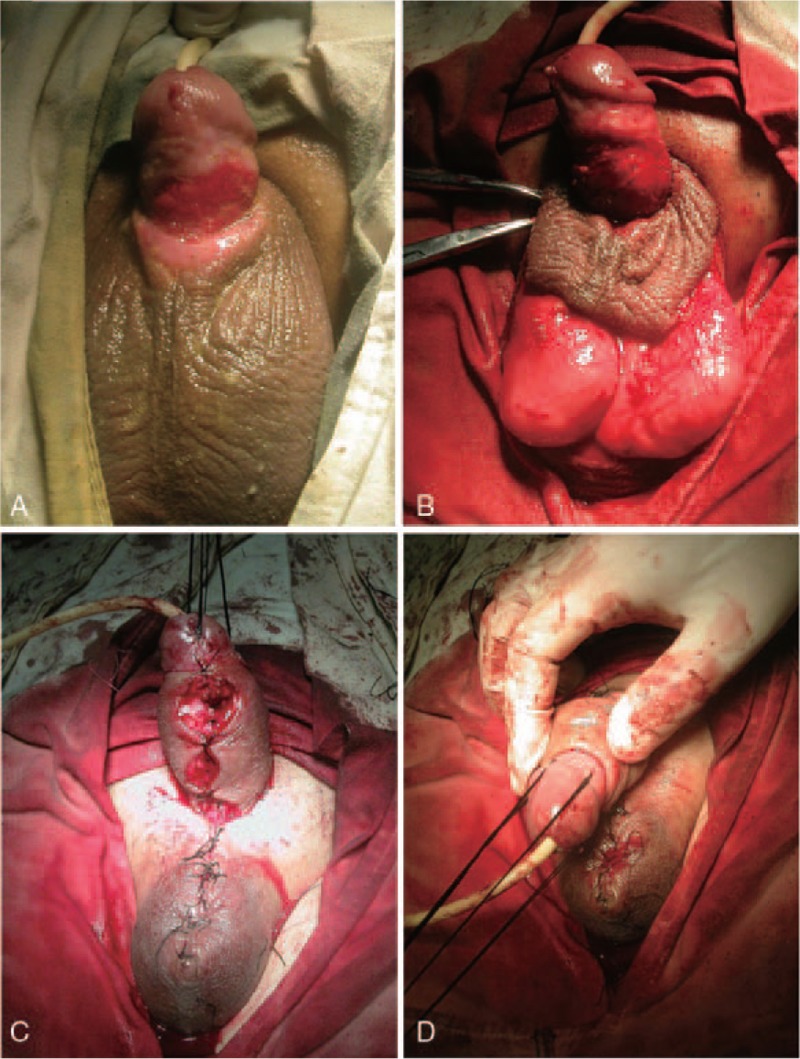
Images from the reconstruction operation. (A) Preoperative photograph showing extensive defects of the penile skin. (B) The reverse bilateral anterior scrotal flap, taken from the anterior side of the scrotum. (C) Reversal of the flap and subsequent wrapping around the penis, from the dorsal side to the ventral side. (D) A Z-suture performed on the ventral surface of the penis.

**Figure 3:**
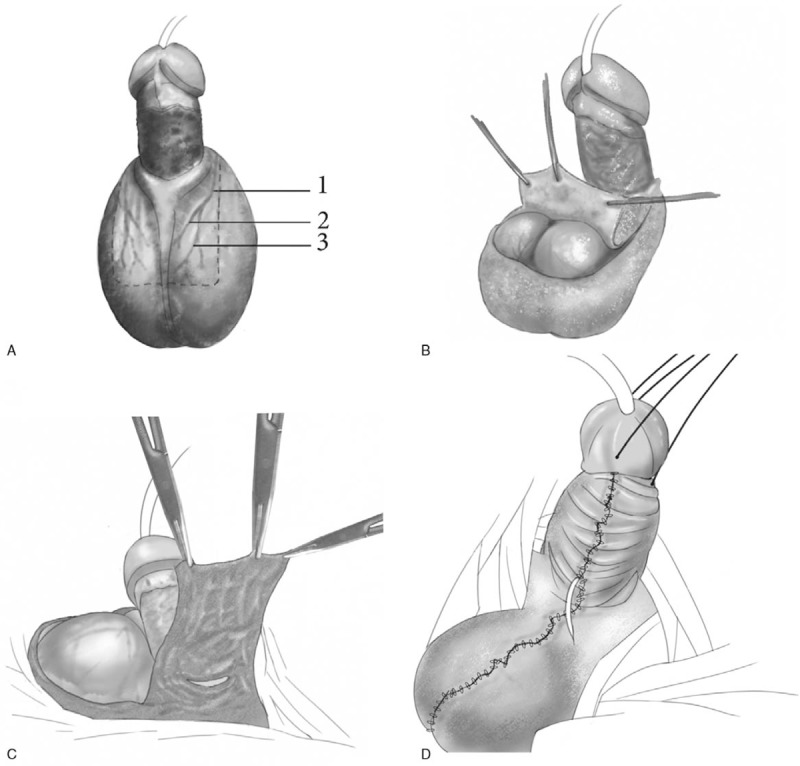
Schematic illustration of the procedure. (A) The flap, pedicled on the bilateral anterior scrotal artery, was taken from the anterior side of the scrotum. The dotted lines represent the skin incision. The flap was supplied by (1) the anterior scrotal artery, (2) the internal branch of the anterior scrotal artery, and (3) the external branch of the anterior scrotal artery. (B) The flap was partially excised and pulled towards the penis. (C) The flap was reversed and wrapped around the penis from the dorsal side to the ventral side. (D) Primary closure of the donor site.

A 31-year-old man was admitted to our care presenting with extensive defects of the penile skin (Fig. 1). On admission, he complained of pain and swelling of the penis and scrotum. He reported a previous circumcision, carried out 20 days before admission to the clinic. Perineal examination revealed a chronic granulating wound with full-thickness skin loss (Fig. 2A). The patient was diagnosed with infection, necrosis, and extensive defects of the penile skin, and subsequently underwent debridement of the penile shaft, in addition to treatment with antibiotics. As a result of the debridement, there was a 5 cm-wide circumferential skin defect on the penile shaft. A reverse bilateral anterior scrotal artery flap was used to reconstruct the penile skin defect (Figs. 2 and 3). The flap used tissue from the anterior side of the scrotum and the dimensions of the flap were 6 cm by 6 cm (Fig. 3A). The scrotal flap was dissected and reversed and then wrapped around the penis from the dorsal side to the ventral side (Figs. 2B, C and 3B, C). A Z-suture was performed to avoid scar contracture and subsequent ventral curvature of the penis (Fig. 2D). Stay sutures were stitched into the tunica albuginea to reduce scarring and penis retraction (Fig. 3D).

**Figure 4:**
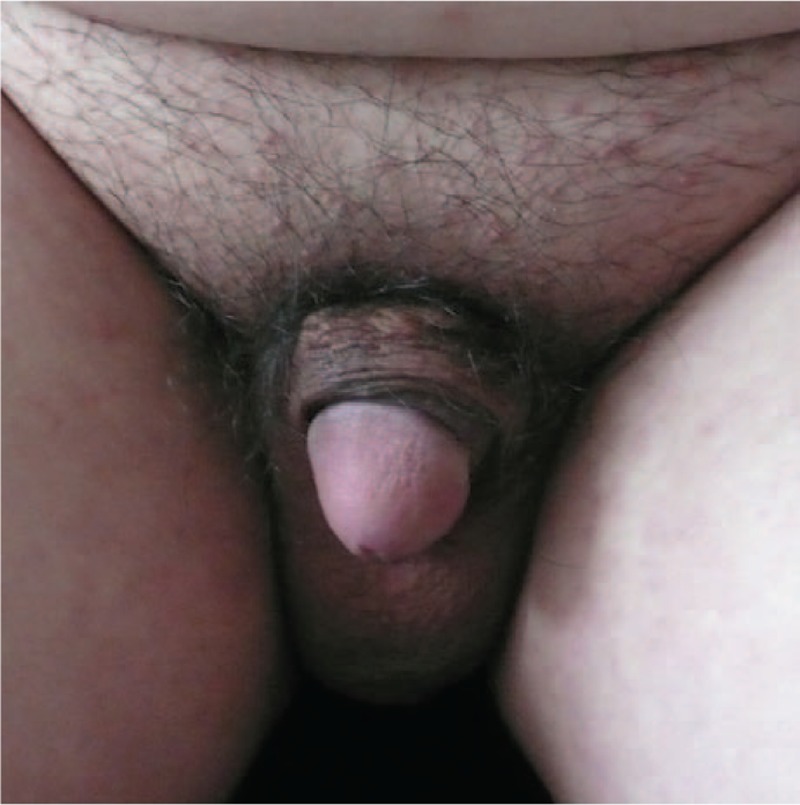
Postoperative photograph of the penis and scrotum showing satisfactory aesthetic outcome (2-year follow-up).

**Figure 5:**
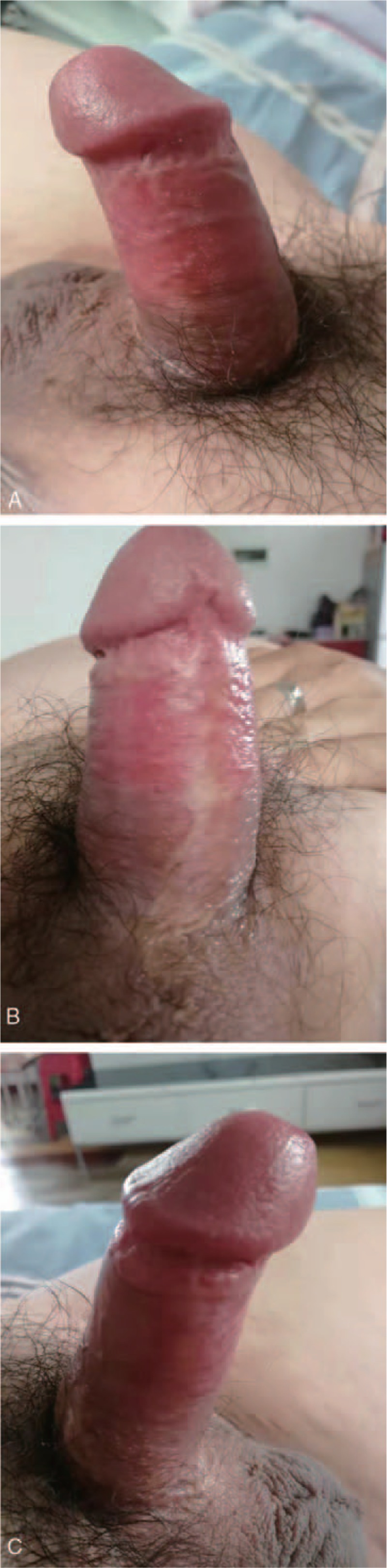
Postoperative photograph of the penis and scrotum showing satisfactory aesthetic outcome (10-year follow-up).

The scrotal skin flap tissue survived well. The patient healed without adverse events and was able to have normal intercourse 2 months after the operation. A 2-year follow-up showed favorable aesthetic and functional results (Fig. 4). Excellent outcomes were noted at the 10-year follow-up (Fig. 5). The patient provided full informed consent for the publication of this case report.

## 3 Discussion

Circumcision is one of the most commonly practiced surgeries in history. Nearly 30% of the world's male population aged 15 years or older are circumcised.^[7,8]^ Defects of the entirety of the penile skin, a rare complication of circumcision, can cause severe physiological disorders and psychologic distress.^[9,10]^ Complete resurfacing of the penile shaft has proved difficult. Penile resurfacing not only requires adequate cosmetic results, but also requires the maintenance or restoration of erectile function, tactile sensibility, and sexual satisfaction. A wide range of medical treatments has been employed to repair defects of the penis. Repair for disorders such as adult-acquired buried penis, where the penis is buried in the scrotum or in the suprapubic region, can be performed, but treatments can cause prolonged aesthetic issues and issues relating to hygiene and sexual function.^[11]^ The results of skin grafting, the most commonly performed penile surgery, can cause trauma during sexual intercourse, and contracture of the graft commonly occurs as a delayed complication.^[12,13]^ Axial pattern skin flaps, such as the deep inferior epigastric perforator (DIEP) flap, the groin flap, the lower abdominal flap, and the tensor fascia lata flap, all present the problem of giving the penis a bulky appearance.^[14]^ By contrast, the advantages of using the scrotal skin as a flap include its lack of subcutaneous fat, high elasticity, similar thickness to penile skin, and rich blood supply.^[15,16]^ The use of scrotal flaps for the coverage of extensive penile defects has not been extensively reported. Fakin et al reported that a bipedicled anterior scrotal flap can cover to 65% of the penile shaft during reconstruction of the penile skin.^[5]^ In this case report, we describe the successful reconstruction of the entire penile shaft using a reverse bilateral scrotal anterior flap. Furthermore, local 1-stage reconstruction minimized the number of surgical procedures required to achieve a suitable aesthetic outcome. The Z-suture on the ventral side was scarcely visible and secondary contraction and scarring could be avoided with the use of the Z-suture. No complications were encountered during the 2-year follow-up period. The patient reported normal erectile function and the ability to perform intercourse, which shows the feasibility and effectiveness of this method.

The scrotal anterior flap has a dual blood supply. Branches of the external and internal pudendal arteries connect with each other and form the blood supply system of the scrotal wall.^[9]^ Anatomical findings and clinical applications have shown that the scrotal flap is thin, versatile, easy to harvest and is generally anatomically consistent between patients.^[17]^ This relatively simple and timesaving procedure is performed in 1 stage; thus, high economic, social, and personal costs are avoided. Moreover, as the scrotal flap contains sensory branches from the ilioinguinal nerve, the use of this tissue appears to contribute markedly to long-term erectile function and sexual satisfaction. Therefore, the advantages of this surgical technique include: the use of easily obtainable tissue from the scrotum, with a similar color, thickness, and texture as the penile skin; the need to perform only a single procedure for full reconstruction; a higher degree of tissue survivability; and the preservation of the sensitivity and function of the penis.

## 4 Conclusion

In conclusion, our report suggests that the reverse bilateral anterior scrotal artery flap is suitable for the repair of complete skin defects of the penis and provides substantial benefits to the patient due to satisfactory cosmetic and final functional results. Further studies will be required to confirm the reliability of this method across a larger cohort of patients.

## Author contributions

**Conceptualization:** Wenrui Qu

**Investigation:** Qing-Guo Gao

**Methodology:** Wenrui Qu

**Writing – original draft:** Qing-Guo Gao

**Writing – review & editing:** Wenrui Qu

Wenrui Qu ORCID: 0000-0002-0178-3698
